# SEC14 Phospholipid Transfer Protein Is Involved in Lipid Signaling-Mediated Plant Immune Responses in *Nicotiana benthamiana*

**DOI:** 10.1371/journal.pone.0098150

**Published:** 2014-05-20

**Authors:** Akinori Kiba, Ivan Galis, Yuko Hojo, Kouhei Ohnishi, Hirofumi Yoshioka, Yasufumi Hikichi

**Affiliations:** 1 Laboratory of Plant Pathology and Biotechnology, Faculty of Agriculture, Kochi University, Nankoku, Kochi, Japan; 2 Institute of Plant Science and Resources, Okayama University, Kurashiki, Japan; 3 Research Institute of Molecular Genetics, Kochi University, Nankoku, Kochi, Japan; 4 Laboratory of Defense in Plant-Pathogen Interactions, Graduate School of Bioagricultural Sciences, Nagoya University, Chikusa-ku, Nagoya, Japan; Agriculture and Agri-Food Canada, Canada

## Abstract

We previously identified a gene related to the *SEC14*-gene phospholipid transfer protein superfamily that is induced in *Nicotiana benthamiana* (*NbSEC14*) in response to infection with *Ralstonia solanacearum.* We here report that *NbSEC14* plays a role in plant immune responses via phospholipid-turnover. *NbSEC14*-silencing compromised expression of defense–related *PR-4* and accumulation of jasmonic acid (JA) and its derivative JA-Ile. Transient expression of *NbSEC14* induced PR-4 gene expression. Activities of diacylglycerol kinase, phospholipase C and D, and the synthesis of diacylglycerol and phosphatidic acid elicited by avirulent *R. solanacearum* were reduced in *NbSEC14*-silenced plants. Accumulation of signaling lipids and activation of diacylglycerol kinase and phospholipases were enhanced by transient expression of *NbSEC14*. These results suggest that the NbSEC14 protein plays a role at the interface between lipid signaling-metabolism and plant innate immune responses.

## Introduction

Plants have evolved innate immune responses to detect and respond quickly to foreign infections [1]. Plants use transmembrane pattern recognition receptors (PRRs) that recognize pathogen-associated molecular patterns (PAMPs) at the cell surface. Plants perceive bacterial flagellin, EF-Tu, and fungal chitin oligomers through their cognate receptors FLS2, EFR, and CERK1, respectively [2], [3]. Plants also recognize avirulent gene products as pathogen infections by polymorphic receptors typically containing nucleotide-binding leucine-rich repeat resistance (R) proteins [4].

After recognition events, intracellular signaling cascades, such as changes in ion fluxes, cytoplasmic Ca^2+^ levels, oxidative burst, protein phosphorylation, and the production of stress-related hormonal substances, are required for the establishment of plant immune responses [5], [6]. The increase in Ca^2+^ concentration and activation of Ca^2+^-dependent protein kinases induces an oxidative burst in the potato after PAMPs recognition [7]. The generation of reactive oxygen species (ROS) and nitric oxide (NO) is also implicated in defense-related gene expression mediated by both PRRs and R proteins [8.9.10]. In *Nicotiana* plants, members of the mitogen activated protein kinase (MAPK) family, SIPK, WIPK, and NTF6, are involved in defense induction in response to PAMPs, INF1 and HWC [11], [12]. Both WIPK and SIPK are also sufficient to induce *N*-gene mediated resistance to the tobacco mosaic virus [13]. In tomato, LeMKK2, LeMKK3, and LeMPK3 are required for Pto-mediated resistance against *Pseudomonas syringae* pv. *tomato* carrying AvrPto [14]. In *Arabidposis* plants, members of the MAPK family, MPK3 and MPK6, are implicated in PRRs and R protein-mediated defense responses [15], [16]. Plant defense responses are also controlled by a complex, interconnected signaling network that includes the hormones salicylic acid (SA), jasmonic acid (JA), and ethylene (ET) with antagonistic interaction of the JA and SA signaling pathways [17]. In *Arabidopsis thaliana*, an SA-dependent cascade is critical for biotrophic immune responses against *Pseudomonas syringae* pv. *tomato* DC3000. In contrast, ET/JA pathways are required for necrotrophic resistance against *Alternaria brassicicola* [18].

Phospholipids-based signaling cascades are common signal transduction mechanisms during plant immune responses. Phospholipases are activated during defense signal transduction. For example, induction of phospholipase D genes (*PLD*) occurs after elicitor treatment of tomato cells [19]. Similarly, treatment of *N*-acetyl chitooligosaccharide elicitor could induce rapid activation of PLD, resulting in the accumulation of phosphatidic acid (PA) in rice cells [20]. An avirulent strain of *Xanthomonas oryzae* induced the expression of both phospholipase C (*PLC*) and *PLD* genes in rice [21]. Isoforms of tomato *PLC* are required for *Cf-4*-dependent immune responses, whereas *SlPLC4* and *SlPLC6* are required for general immune responses [22]. Among the phospholipids, PA has been shown as intracellular signaling molecule leading to plant immune responses. In tomato suspension-cultured cells, PA and diglycerol pyrophosphate accumulate in response to a xylanase elicitor [23]. PA also accumulates in tomato cells in response to a race-specific Avr4 elicitor in a *Cf-4* dependent manner [24].

Phospholipid metabolism and signaling are important in plant immune responses, although the molecular regulatory mechanisms of phospholipid synthesizing enzymes have remained elusive. Previously, we identified a gene related to the *SEC14*-gene superfamily from *Nicotiana benthamiana* (*NbSEC14*). *NbSEC14* rescued temperature-sensitive growth mutant of sec14 in yeast, and NbSEC14 protein showed phospholipid transfer activity. Moreover, acceleration of disease development of bacterial wilt and growth of *R. solanacearum* were observed in the *NbSEC14*-silenced plants [25]. SEC14 protein belongs to the large yet under-characterized Sec14-protein superfamily (1550 proteins) originally isolated from *Saccharomyces cerevisiae* [26], [27], [28]. SEC14 protein functions as phospholipid transfer protein and acts in the phospholipid metabolism and phosphoinositide signaling pathways involved in diverse cell functions [29], [30], [31]. However, there is no information about role of SEC14 phospholipid transfer protein in plant immune responses. In this study, we analyzed the role of *NbSEC14* phospholipid transfer protein in plant immune responses in *N. benthamiana*. In addition, we also discuss a possible relationship between phospholipid turnovers and NbSEC14 protein that leads to plant immune responses.

## Materials and Methods

### Plant Materials

*Nicotiana benthamiana* was grown in a plant growth room as described before [32].

### Bacterial Isolates, Culture Conditions, and Inoculation

Bacterial strains used in this study are listed in Table S1. *Ralstonia solanacearum* strains 8107 (Rs8107), *Pseudomonas cichorii* SPC9018 were cultured in PY medium containing 20 µg/mL rifampicin. The density of bacterial suspension was adjusted to 1.0×10^8^ CFU/mL and inoculated by leaf infiltration as described in Maimbo *et al.,* [32].

### Primers and Plasmids

Primers and plasmids used in this study are listed in Tables S2 and S3, respectively.

### RNA Isolation

Total RNA was prepared from *N. benthamiana* leaves with RNAiso (Takara Bio, Shiga Japan) according to the manufacturer’s procedure. RNA samples were then treated with DNase I (RNase-free; Takara) to degrade contaminating genomic DNA as described previously [32].

### Quantitative Real Time PCR

Quantitative real time PCR was performed by the method described in Maimbo *et al.,* [32]. Reverse transcription was performed with 1 µg total RNA using PrimeScript RT reagent Kit (Takara), and qRT-PCR with 20 µL of a reaction mixture containing 1 µL of cDNA template, and 10 pM of the respective primers using the SYBR GreenER qPCR Reagent System (Invitrogen, Tokyo Japan) and an Applied Biosystems 7300 real time PCR instrument. Cycling parameters were the same for all primers: an initial 50°C for 2 min and 95°C for 10 min, followed by 40 cycles of 95°C for 10 s and 60°C for 1 min. Melting curve runs were performed at the end of each PCR reaction to verify the specificity of primers by the presence of a single product. The expected single DNA product and its molecular weight were verified by agarose gel electrophoresis. We also checked the sequence of amplified DNA products by direct sequencing with an upper primer. Relative quantification of gene expression was performed according to the instructions for the Applied Biosystems 7300 real-time PCR system using the comparative cycle threshold [Ct] method for the calculation of Qty value. All values were normalized to the expression values of the actin gene used as an internal standard in each cDNA stock. Expression analyses were performed with at least two biological replications to ensure that expression patterns were reproducible, and representative data are presented. Standard deviations and differences between expression ratios of non-treated controls and other samples were tested for statistical significance using the Student t-test.

### Vector Constructs and Seedling Infection for Virus-induced Gene Silencing

A 389-bp cDNA fragment of the 3′-terminal region of *NbSEC14* was used for Virus-Induced Gene Silencing (VIGS) experiments as described previously [25]. Construct for *NbCoi1*-silencing was prepared as described previously [33]. Plasmid pPVX201 with no insert was used as a control. All binary plasmids were transformed into *A. tumefaciens* strain GV3101 [34] and inoculated into *N. benthamiana* leaves as described previously [32]. Specificity of *NbSEC14*-silencing was tried to confirm by *NbSEC14*-silencing with two different parts of *NbSEC14* cDNAs (Figure S1) and Southern blot analysis with cDNA fragment (sec14P1) as probe. According to results of Southern blot, we could observed single band, suggesting specific silencing of NbSEC14 [25]. However, we found out two different contigs (Nbs00015170g0012.1 and Nbs00058777g0001.1) in Sol Genomics database (http://solgenomics.net/). Because they have more than 92% nucleotide identities with NbSEC14, we judged that silencing might affect all members of NbSEC14 family, and we therefore observed an overall effect of gene silencing of NbSEC14 family on plant immune responses.

### Plasmid Construction for *Agrobacterium*-mediated Transient Expression

A full-length open reading frame (ORF) of *NbSEC14* with a FLAG tag was amplified with secORF-S and secFlag-A primers using pGEMNbSEC14 [25] as a template, and the PCR product cloned into pGEMT-Easy vector (pGEMNbSEC14Flag). pGEMNbSEC14Flag was then digested with *Bam*HI and *Sac*I (Takara), and insert was cloned into the pBI121 vector (CLONTHEC, Tokyo, Japan) digested with the same enzymes. The final construct was designated pBI-NbSEC14. For agroinfiltration experiments, we also used the binary vector p35S-INF1 [35]. The binary vector p35S-GUS containing the GUS gene [36] was used as a control. These binary plasmids were transformed into *A. tumefaciens* strain GV3101, and inoculated into *N. benthamiana* leaves as described previously.

### Phytohormone Analysis

Phytohormone contents were measured by the method described previously [37]. Extracted samples were subjected to measurement on a triple quadrupole LC-MS/MS 6410 (Agilent Technologies, USA) equipped with a Zorbax SB-C18 column [2.1 mm id×50 mm, (1.8 µm), Agilent Technologies]. Hormone amounts were calculated from the ratio of endogenous hormone peak and known amount of internal standards spike, and related to actual fresh mass of the samples used for extraction.

### Protein Analysis

Preparation of crude protein fractions and protein analysis were performed as described [38]. Crude protein fractions isolated from *N. benthamiana* leaves were separated by 12.5% SDS-PAGE and then electroblotted onto polyvinylidene difluoride membranes (Bio-Rad Labs., Hercules, CA). The blots were subjected to western blot analyses with a monoclonal antibody raised against the Flag-tag sequence (Sigma-Aldrich, Tokyo, Japan). Cross-reacting proteins were visualized with a goat alkaline phosphatase secondary antibody (BioRad) conjugated with 5-bromo-4-chloro-3-indolylphosphate and nitroblue tetrazolium (Nacalai Tesque, Kyoto, Japan). Equal loading of protein fractions was estimated by Coomassie brilliant blue staining of Rubisco large subunit.

### *In vivo* [^32^P]Phospholipid Labeling

*N. benthamiana* leaves were detached and leaf petioles were dipped in water containing 0.59 Mbq carrier-free [^32^P]orthophosphate (Muromachi Chemical, Tokyo, Japan) and incubated at 25°C for 12 h.

### Phospholipid Extraction, Separation and Analysis

Total lipids were extracted in CHCl_3:_MeOH:HCl (50∶100∶1, v/v/v) according to the method described in Munnik *et al.,* [38]. Total lipid extracts were dried by vacuum centrifugation, dissolved in CHCl_3_, and separated by thin layer chromatography (TLC) with Silica 60 TLC plate (Merck, Darmstadt, Germany) using three different solvents. An alkaline TLC [CHCl_3_:MeOH: 25% NH_4_OH:H_2_O (45∶35∶2∶8, v/v/v/v)] was used to isolate phosphatidylethanolamine (PE), phosphatidylglycerol (PG) and PI, and an acidic TLC [CHCl_3_:CH_3_COCH_3_:MeOH:CH_3_COOH:H_2_O (40∶15∶14∶13∶7.5, v/v/v/v/v)] for PC, as described by Munnik *et al*., [39], [40]. An ethyl acetate solvent system [the organic upper phase of ethyl acetate/isooctane/formic acid/H_2_O (13∶2∶3∶10, v/v/v/v)] was used to separate PA from the other phospholipids [40]. Radiolabelled lipids were visualized and quantified by autoradiography, with densitometry scans performed on a GE Storm 860 with ImageQuant TL (GE Healthcare, Tokyo, Japan). Relative amount of phospholipid was calculated as relative to the none-treated sample.

### DAG Quantification by DAG Kinase Reaction

Quantification of DAG levels was performed by measuring DAG ^32^P-phosphorylation using *Escherichia coli* DAG kinase as described by Zien *et al.,* [41], with slight modifications. First, leaf-extracted lipids were dried for a short time period under nitrogen. Second, micelles (20 µL) containing 7.5% octyl-L-D-glucopyranoside (Nacalai) and 20 mg mL^−1^ of 1,2-dioleoyl-sn-glycero-3-phosphoglycerol (Funakochi, Tokyo, Japan) were added to the plant lipid mixture. Third, a reaction mixture (50 µL) containing 10 mM imidazole (pH 6.6), 10 mM LiCl, 25 mM MgCl_2_, 2 mM EDTA, and 19.4 µL of dilution buffer containing 10 mM imidazole (pH 6.6), 1 mM diethylenetriaminepentaacetic acid. This was followed by the addition of 10 µL of 2 mM ATP with 2.5 µCi of γ-^32^P-ATP to the previous mix. Fourth, the reaction of DAG ^32^P-phosphorylation was started by the addition of 100 units of *E. coli* DAG kinase (Sigma) and incubated at 25°C for 1 h. Lipids were extracted with the procedure described by Bligh and Dyer [42]. The organic phase was dried down under nitrogen, resuspended in chloroform, spotted on TLC plates, and separated with an ethyl acetate solvent system. Radio-labeled PA were visualized by autoradiography, and densitometry scans of autoradiograms were performed using a GE Storm 860 and ImageQuant TL (GE Healthcare). DAG content was normalized to leaf fresh weight.

### Assay for Phospholipase Enzymatic Activities

PLD activity was measured as the production of phosphatidylbutanol (PBut), as described previously [43], [44]. Briefly, *N. benthamiana* leaves were prelabeled with ^32^P for 12 h, and infiltrated with Rs8107 suspension with 0.25% n-butanol. Incubations were stopped and lipids extracted as described above. ^32^P-labelled PBut was separated by an ethyl acetate TLC system, and its radioactivity visualized and quantified as described above. Relative phospholipase activity was calculated relative to the absolute PVX at time 0.

In vitro phosphoinositide-specific phospholipase C activity was measured by the hydrolysis of ^3^ H-PIP_2_ as described previously [45], [46]. Briefly, total protein fractions were incubated at 25°C for 30 min in Tris-Malate (pH 6.0) containing 10 µM CaCl_2_ and 200 µM PIP_2_ spiked with 0.86 KBq ^3^H-PIP_2_. Reactions were stopped by addition of chloroform:methanol (2∶1, v/v). 0.2 M HCI. Samples were centrifuged at 10,000×*g*, and radioactivity in the water-soluble upper phase counted with a liquid scintillation system.

### Statistical Analysis

Statistical analysis was carried out using *t*-test.

## Results

### NbSEC14 Protein Regulates the Expression of Defense-related Genes

**Figure 1:**
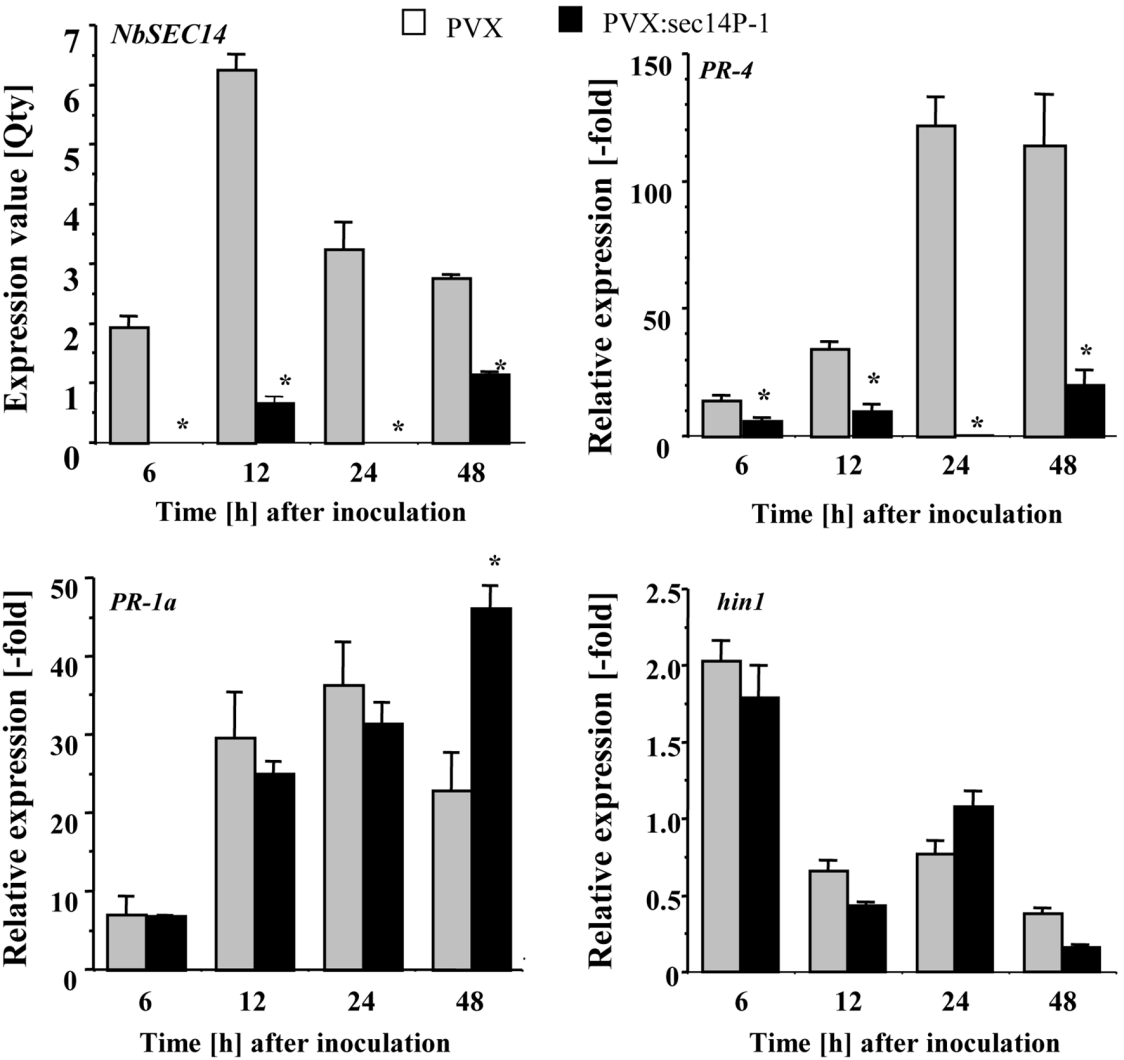
Virus-induced gene silencing of *NbSEC14* and its effect on the expression of defense-related genes. Total RNA was isolated from control (PVX; gray box) and *NbSEC14*-silenced leaves (PVX:sec14P1; black box) infiltrated with avirulent Rs8107. Expression values of *NbSEC14* are expressed as [Qty] after normalization with actin. Relative expression of *PR-4, PR-1a,* and *hin1* transcripts were normalized with actin and calculated as relative to the non-treated control. Values represent the means and SD from triplicate experiments. Asterisks denote values significantly different from empty PVX controls (*; *P*<0.05).

**Figure 2:**
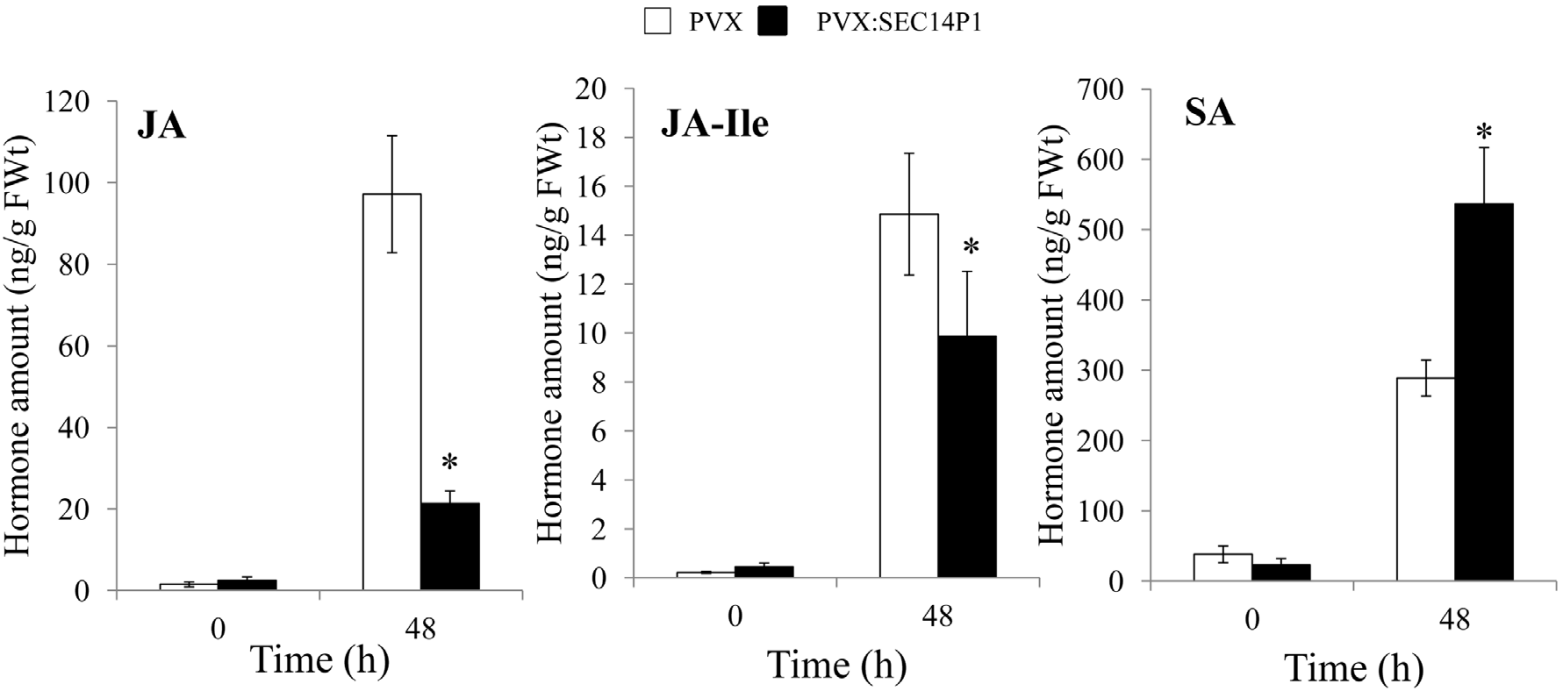
Effect of silencing of *NbSEC14* on plant hormone contents. Control (PVX) and *NbSEC14-*silenced (PVX:sec14P1) *N. benthamiana* plants were infiltrated with Rs8107. Plant hormone contents of jasmonic acid (JA), jasmonoyl-L-isoleucine (JA-Ile) and salicylic acid (SA) were determined at designated time points by LC-MS/MS. Values represent the means and SD from triplicate experiments. Asterisks denote values significantly different from empty PVX controls (*; *P*<0.05).

*NbSEC14*-silencing was carried out independently by *NbSEC14*-silencing with two different parts of the *NbSEC14* cDNA (P1, P2), showing very similar effect on immune responses in both cases (Figure 1, Figure S1). Therefore, we only used SEC14P1 fragment for further analysis. To determine the role of NbSEC14 in plant innate immunity, we focused on characteristic immune responses, including hypersensitive response (HR), salicylic acid (SA)-dependent and jasmonic acid (JA)-dependent signaling pathways. We could not observe any visible differences in HR induction in both control and *NbSEC14*-silenced plants (Figure S2). In control leaves (empty vector VIGS) inoculated with Rs8107, expression of *NbSEC14* and *PR-4*, a marker gene for JA signaling, showed peaks of expression at 12 and 24 hours after inoculation (HAI), respectively, but *PR-4* transcript levels were greatly reduced in *NbSEC14-*silenced leaves. In contrast, expression of *PR-1a*, a marker gene for SA signaling, was rather enhanced in the silenced plants 48 HAI with Rs8107. In the case of *hin1*, the HR-related gene, the expression was much less affected by *NbSEC14-*silencing (Figure 1). These results suggested a possible involvement of JA in NbSEC14-related immune responses. Then, we confirm JA and JA-Ile contents as well as SA content. As shown Figure 2, accumulation of JA and JA-Ile was observed in control plants challenged with Rs8107, whereas significant reduction of both hormone contents was observed in NbSEC14-silenced plants. In contrast, we could detect SA accumulation and hyper-accumulation of SA was observed in the silenced plants compared to control plants.

**Figure 3:**
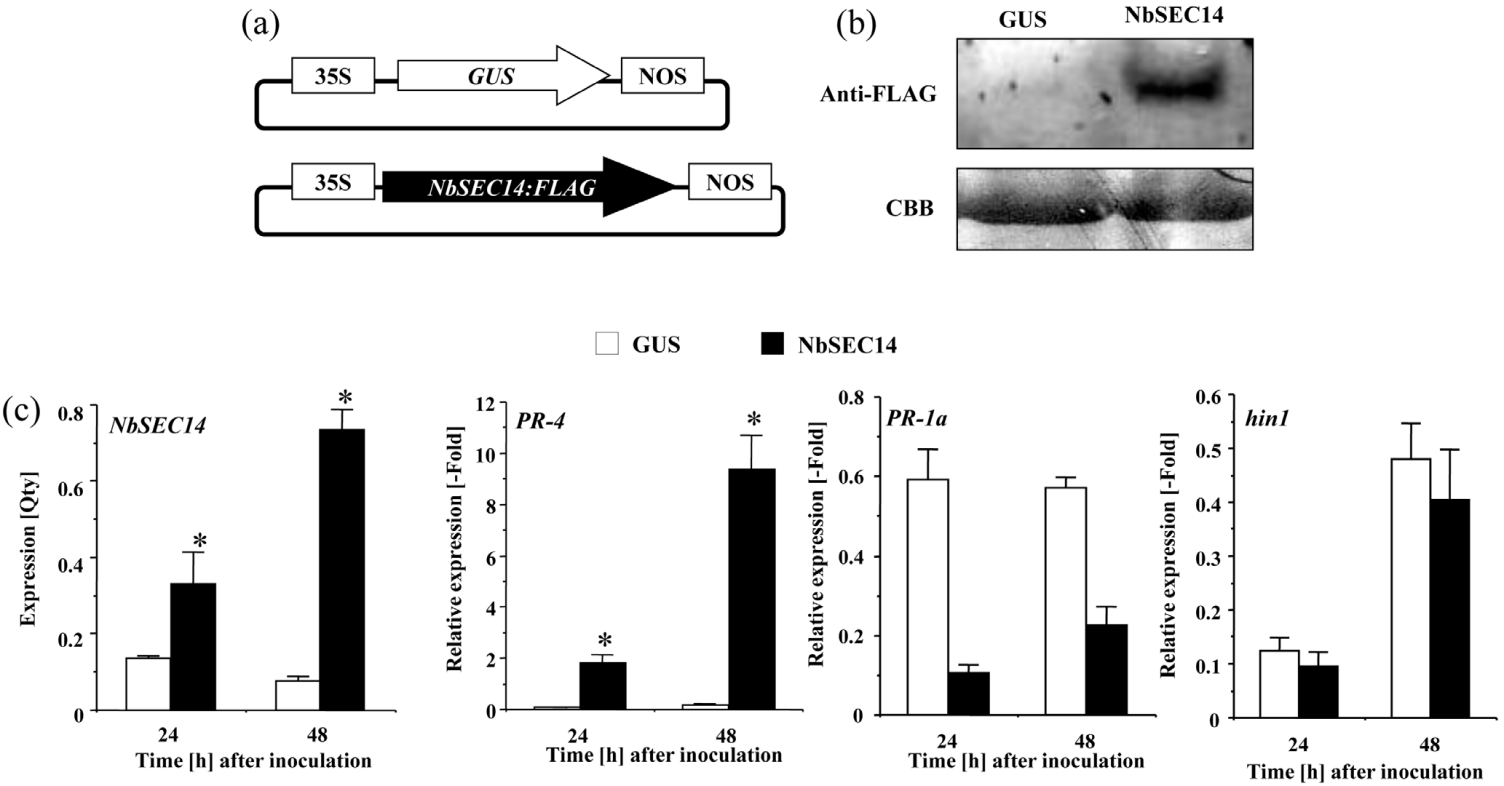
Transient expression of *NbSEC14* and induction of defense-related genes expression. (a) Diagram illustrating constructs used for transient expression of *NbSEC14*. (b) Total protein extracts were prepared from *N. benthamiana* leaves inoculated with *Agrobacterium* carring *NbSEC14* (NbSEC14) and GUS control (GUS), and separated by SDS-PAGE. Equal amounts of loaded protein fractions were estimated by Coomassie Brilliant Blue staining of the Rubisco large subunit (CBB), and NbSEC14 protein: FLAG detected by western blot using monoclonal antibodies raised against the Flag-tag (Anti-Flag). (c) Total RNA was isolated from *N. benthamiana* leaves inoculated with GUS and *NbSEC14* expressing *Agrobacterium*. Relative expression of *PR-4, PR-1a,* and *hin1* transcripts were normalized with actin and calculated as relative to the non-treated control. Values represent the means and SD from triplicate experiments. Asterisks denote values significantly different from GUS-expressing controls (*; *P*<0.05).

Because silencing of *NbSEC14* reduced *PR-4* expression, but not *PR-1a* and *hin1*, we tested the effect of the transient expression of *NbSEC14* in *N. benthamiana* leaves on the expression of these genes (Figure 3a). qRT-PCR and western blot analysis confirmed a higher level of overexpressed *NbSEC14*, up to 8 times more relative to β-glucuronidase (*GUS*) gene-expressing control leaves at 48 hours (Figure 3b, c). Expression of *PR-1a* was significantly reduced, but *hin1* expression was not affected. In contrast, *PR-4* expression was significantly elevated in *NbSEC14*-overexpressing plants compared to control plants (Figure 3d). To strengthen our hypothesis of NbSEC14 interacting with JA signaling, we focused on *NbCoi1* gene, which encodes F-box protein and have been well know as positive regulator of JA signaling [47]. Then, we examined the effect of *NbCoi1*-silencing on *PR-4* induction by transient expression of *NbSEC14*. A strong reduction of *PR-4* expression controlled by NbSEC14 in *NbCoi1*-silenced plants was observed (Figure S3). Taken together, NbSEC14 protein may associate with the expression of defense-related genes related to JA-dependent pathway.

### NbSEC14 Protein Regulates Phospholipid Signaling during Plant Immune Responses

**Figure 4:**
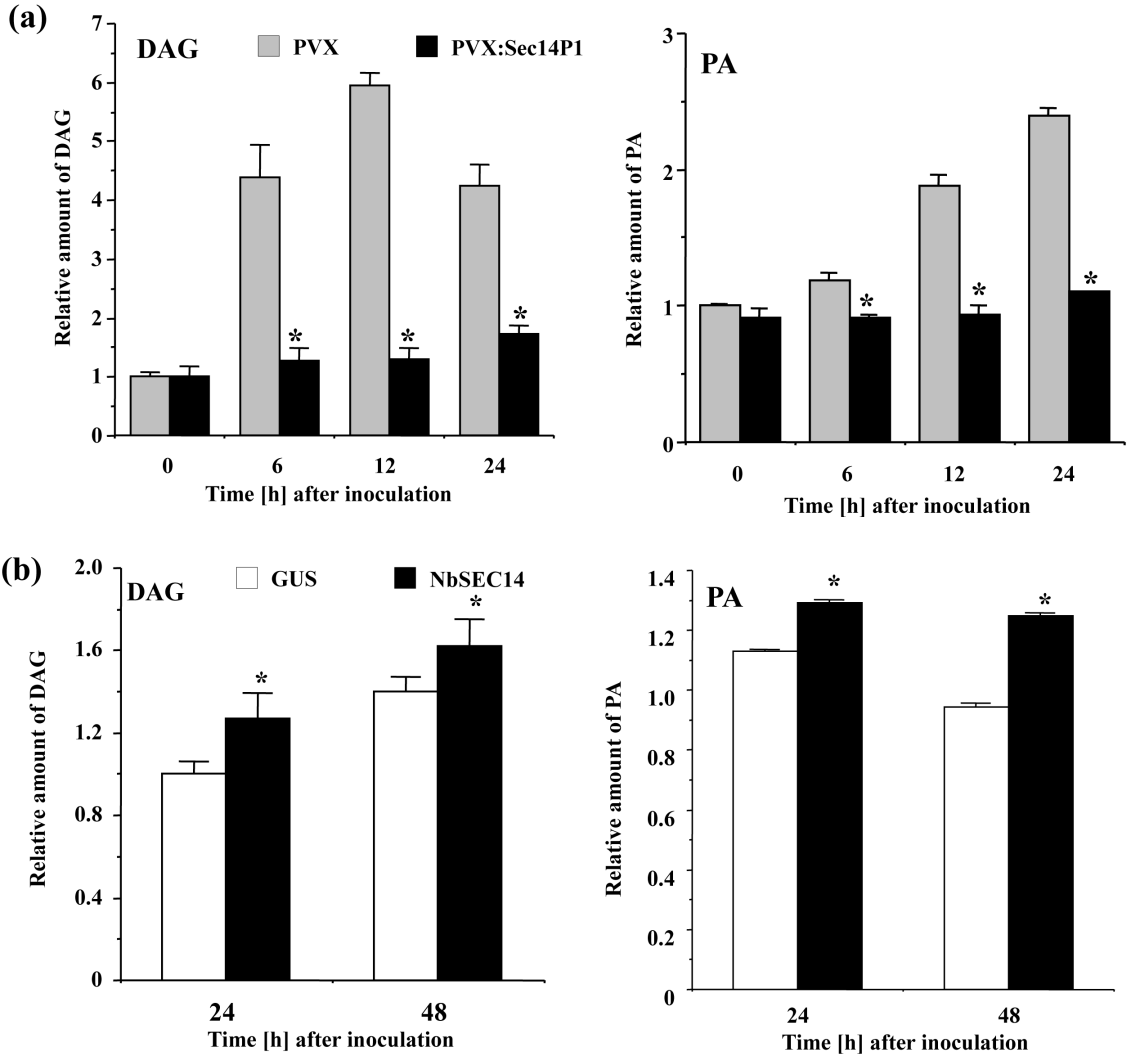
NbSEC14 affects phospholipids contents. Control (PVX; gray box) and *NbSEC14*-silenced (PVX:Sec14P1; black box) leaves were pre-labeled with ^32^Pi for 12 h and inoculated with Rs8107. (a) Quantification of DAG and PA in control and *NbSEC14*-silenced leaves after inoculation with Rs8107. Relative amount of phospholipid was calculated as relative to the none-treated sample. (b) Quantification of DAG and PA in *N. benthamiana* inoculated with *NbSEC14* (NbSEC14) and GUS control (GUS) expressing *Agrobacterium*. Values represent the means and SD of the results from triplicate experiments. Phospholipid levels are calculated relative to the absolute non-treated control. Asterisks denote values significantly different from GUS-expressing controls (*; *P*<0.05). Total lipid fractions were separated by ethylacetate TLC for quantification of PA. DAG levels were estimated with DAG kinase system as described in the Materials and Methods.

NbSEC14 protein exhibits phospholipid transfer activities [25], and may be involved in plant immune response. Given these results, we evaluated the role of NbSEC14 protein in plant phospholipid metabolism in relation to its role in immunity. Among the phospholipids, we first focused on signaling phospholipids, diacylglycerol (DAG) and PA, and determined changes of DAG and PA in control and *NbSEC14*-silenced plants challenged with Rs8107. The formation of DAG and PA increased in Rs8107-inoculated control leaves, with peak DAG levels 12 HAI and peak PA levels at 24 HAI, whereas both phospholipid content was significantly reduced in *NbSEC14*-silenced plants (Figure 4a). In addition, *NbSEC14*-expressing plants showed increased DAG (24 HAI) and PA (48 HAI) compared to *GUS*-expressing controls (Figure 4b).

### Regulation of Phospholipase Activities by NbSEC14 Protein

**Figure 5:**
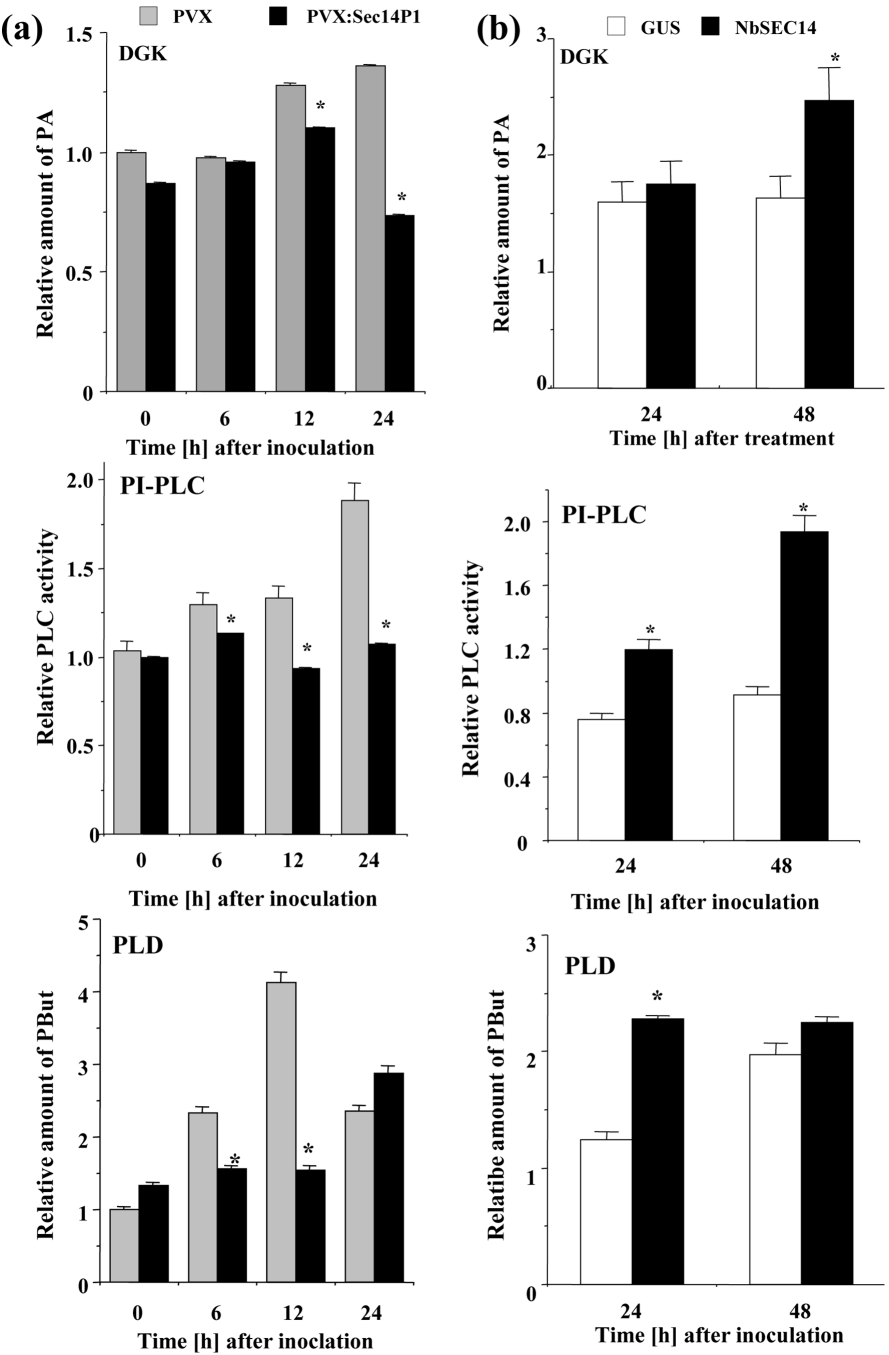
Regulation of phospholipase activities by NbSEC14. (a) Effect of *NbSEC14*-silencing on activities of DGK, PI-PLC and PC-PLD. Control (PVX; gray box) and *NbSEC14*-silenced (PVX:Sec14P1; black box) *N. benthamiana* leaves were inoculated with Rs8107 and incubated at 25°C for indicated times. Phospholipid quantity was calculated relative to the absolute PVX at time 0. (b) DGK and phospholipase activity in *NbSEC14*-expressed plants. Total protein extracts were prepared from *N. benthamiana* leaves inoculated with *NbSEC14* (NbSEC14) and GUS control (GUS) expressing *Agrobacterium*. Phospholipid levels are calculated relative to the non-treated control samples. Values represent the means and SD of the results from triplicate experiments. Asterisks denote values significantly different from controls (0) (*; *P*<0.05).

Diacylglycerol kinase (DGK)-PLC and PLD pathways are the two major metabolic pathways that produce DAG and PA [46]. A supporting pharmacological experiment showed that immune responses might be indeed related to PLC and PLD, since population of Rs8107 was stimulated in the concomitant presence of PLC and PLD inhibitors (Figure S3). We therefore analyzed the relationship between NbSEC14 protein and these phospholipid metabolic enzymes during immune responses. DGK activity was enhanced 12–24 HAI, whereas activation of DGK activity was compromised in *NbSEC14*-silenced plants. *NbSEC14*-silencing blocked the increase in PLC activity at 6–24 HAI induced by Rs8107. *NbSEC14* silencing also blocked the increase in PLD activity, as measured by ^32^P-phosphatidylbutanol production, that occurred from 6–12 HAI (Figure 5a). In contrast, overexpression of *NbSEC14* in *N. benthamiana* increased all DGK, PLC and PLD activities (Figure 5b).

## Discussion

In this experiment, we could observe that *NbSEC14*-silencing caused dramatic changes of signaling phospholipids after plants were challenged with Rs8107 (Figure 4). Among the phospholipids, DAG is well known as signaling phospholipid and is reportedly shown to play a crucial role in the response of tobacco cells to aluminum ions [48]. DAG is likely to act as a signaling molecule in tobacco pollen tubes [49]. PA is also recognized as signaling phospholipid [45]. PA is produced in response to xylanase treatments, and the accumulation of PA induces ROS production and cell death in tomato cells and *Arabidopsis* [50], [51], [52], [53]. Wound-induced PA accumulation causes JA accumulation in *Arabidopsis* plants [54]. Here, we showed that *NbSEC14*-silencing blocked increases in JA contents and JA-dependent *PR-4* genes, but did not affect *hin1* gene and subsequent HR (Figure 1, 2 and Figure S2). Expression of SA-dependent *PR-1a* was rather enhanced in the silenced plants. Transiently increasing NbSEC14 protein enhanced the expression of defense-related *PR-4*, whereas *PR-1a* expression was significantly reduced (Figure 3). In addition, up-regulation of *PR-4* gene expression was reduced in *NbCoi1*-silenced plants (Figure S3). Therefore, these results suggested the direct or indirect relation of NbSEC14 on JA-dependent signaling pathway. *NbSEC14* silencing caused faster growth of avirulent and virulent bacteria, and acceleration of disease development by virulent bacteria, and reduction *PR-4* expression was also observed in the silenced plants in response to virulent bacteria [25]. The competitive interactions between SA and JA, and negative effects of JA on SA have been already described by Pieterse et al. [55] and others. Therefore, NbSEC14 protein may be closely associated with plant immunity related to the JA pathway, with interference of SA signaling by competitive interaction of the JA and SA signaling pathways.

Several plant SEC14-like proteins were reportedly shown to play a key role in the lipid-mediated signaling, and PA, DAG, and phosphoinositides (PIPs) regulate important cellular functions. AtSfh1p transfers phosphatidylinositol (PI) and phosphatidylcholine (PC) *in vitro*, in addition to stimulating intracellular and plasma membrane PIP_2_ in a polarity landmark pattern that focuses membrane trafficking, Ca^2+^ signaling, and cytoskeleton functions at the growing root hair apex [56]. AtPATL1, a novel cell-plate-associated protein, regulates membrane lipid composition (PI and PC) to activate PLD [57]. Ssh1p directly activates PI-3-kinase and PI-4-kinase in response to hyperosmotic stress [58]. Schaaf *et al.*, [59] have shown that Sec14 protein is capable of stimulating the production of PIPs by presenting PI to PtdIns 4-kinase. PIP can then serve as a substrate for a PIP kinase to make another class of lipid signaling molecules, PtdIns-4,5-P_2_ (PIP_2_), and PIP_2_ can then be a substrate for PLC and generate DAG and PA. PC can be hydrolyzed by either PLD to generate PA [60] or by PC-PLC to generate phosphocholine and DAG in plants [61]. In animals, PLC produces DAG as a second messenger [60]. *NbSEC14*-silencing reduced PLC and PLD activity in response to Rs8107 inoculation, whereas transient expression of *NbSEC14* activated both enzyme activities (Figure 5). We could observe drastic changes of signaling phospholipids in *NbSEC14*-silenced plant as well as *NbSEC14*-expressing plants (Figure 4). NbSEC14 protein transfers PC and PI in vitro [25]. Unfortunately, although we could not determine actual substrate(s) for NbSEC14 protein in planta, we speculated that NbSEC14 protein affected lipid signaling-mediated plant immune systems in *Nicotiana* through PLC and PLD activities.

The PLC and PLD pathways are crucial in plant defense. Indeed, treatment of an *N*-acetyl chitooligosaccharide elicitor could induce rapid activation of PLD and the accumulation of PA, increasing elicitor-responsive genes as well as phytoalexin biosynthesis in rice cells [20]. Phytoalexin production induced by treatment with the glycopeptide elicitor from *Mycosphaerella pinodes* is mediated by a PIP_2_-PLC pathway [61], [62]. Pharmacological experiments suggested that PLC and PLD might have an important role in the plant immune response against *R. solanacearum* (Figure S3). Inversely, *NbSEC14*-silencing did not affect HR induction, but did affect resistance to both virulent and avirulent bacteria (Figure S2) [25]. These results further imply that NbSEC14 protein may influence HR-independent defense via phospholipase-mediated phospholipid metabolism.

In conclusion, we have speculated that NbSEC14 protein may influence on PLC and PLD activities, as well as downstream PA and DAG production, associated with innate immune responses during bacterial infections (Figure S5). With the capacity of NbSEC14 protein to change the expression of defense-related genes via JA signaling, further studies will be required to clarify the complex mechanism by which NbSEC14 protein is engaged in plant immunity, and to characterize the phospholipases and/or kinases involved in the signaling cascades.

## Supporting Information

Figure S1 **Alignment of** ***NbSEC14*** **with its homologues in** ***N. benthamiana*** **genome, and virus-induced gene silencing of** ***NbSEC14*** **.** (a). Alignment of *NbSEC14* with its homologues in *N. benthamiana* genome. cDNA fragments used for VIGS experiments are shown in gray boxes (Sec14P1) and black box (Sec14P2). Bold characters with underlines show primer sequences used for qRT-PCR (secrtpF and secrtpR). Dashed lines show nucleatide sequences that are not presented. (b) VIGS of *NbSEC14* with Sec14P2 cDNA, and the effect of *NbSEC14* on *NbSEC14* and *PR-4* expression by inoculation with avirulent *R. solanacearum*. Asterisks denote values significantly different from PVX controls (*; *P*<0.05, *t*-test).
(TIF)

Figure S2 **Effect of** ***NbSEC14*** **-silencing on HR induction.** Control and *NbSEC14-*silenced *N. benthamiana* plants were infiltrated with HR-inducible Rs8107, *P. cichorii* (Pc) or *Agrobacterium* harboring 35S-GUS (control GUS) or 35S-INF1 (INF1). (a) Pictures of *N. benthamiana* leaves taken 4 day after infiltration with each bacterium. (b) Control (Control; gray box) and *NbSEC14-*silenced (VIGS; back box) *N. benthamiana* plants were infiltrated with Rs8107, *P. cichorii* (Pc) or *Agrobacterium* harboring 35S-INF1 (INF1). Cell death was determined by Evans blue staining (OD600 nm disk^−1^).
(TIF)

Figure S3 **Role of jasmonic acid pathway in** ***NbSEC14*** **-induced** ***PR-4*** **genes expression.** Total RNA was isolated from control (PVX), *NbCoi1-*silenced (Coi) *N. benthamiana* leaves inoculated with GUS and *NbSEC14* expressing *Agrobacterium.* Relative expression of *NbSEC14* and *PR-4* transcripts were normalized with actin and calculated as relative to the GUS-expressing control. Values represent the means and SD from triplicate experiments. Asterisks denote values significantly different from empty vector (PVX)-expressing controls (*; *P*<0.05).
(TIF)

Figure S4 **PLC and PLD activity is required for defense responses in** ***N. benthamiana*** **.** (a) Schematic of phospholipase-dependent reactions in plant cells. (b) *N. benthamiana* leaves were infiltrated with Rs8107 (10^8^ CFU ml^−1^) in the absence (Mock) or concomitant presence of 50 µM 1-[6-[((17β)-3-Methoxyestra-1,3,5[10]-trien-17-yl)amino]hexyl]-1H-pyrrole-2,5-dione (U73122; PLC inhibitor) and 0.1% normal-butanol (n-ButOH; PLD inhibitor) or 50 µM 1-[6-((17b-3-Methoxyestra-1,3,5(10)-trien-17-yl)amino)hexyl]-2,5-pyrrolidinedione (U73343) +0.1% 2-butanol (2-ButOH)(inactive analogue) (Kirik and Mudgett 2010), and bacterial population was determined by plating at specified time points. Values are means of four replicate experiments with SD. Asterisks denote values significantly different from control palnts (*; *P*<0.05). (c) Effect of inhibitors on in vitro growth of Rs8107. The bacteria were cultured in PY medium in the absence or presence of inhibitors (50 µM U73122+0.1% n-ButOH or 50 µM U73343+0.1% 2-ButOH). There was no obvious toxicity to bacterial growth. (d) Effect of inhibitors on *PR-4* expression by *NbSEC14-*expression in *N. benthamiana*. GUS (GUS) or NbSEC14-expressing *Agrobacterium* (SEC14) were infiltrated in the absence (Mock) or presence of inhibitors into *N. benthamiana*. Expression values of *PR-4* is expressed as [Qty] after normalization with actin. Values represent the means and SD from triplicate experiments. Asterisks denote values significantly different from GUS-expressing controls (*; *P*<0.05).
(TIF)

Figure S5 **Function of NbSEC14 protein in the induction of plant immunity.** *NbSEC14* is expressed in response to bacterial infections. NbSEC14 protein initiates the binding/transfer of phospholipids, leading to changes in membrane lipid composition and substrate supply for lipid kinases and/or phospholipases. The subsequent generation of phospholipid-derived second messengers regulates other defense-related genes and the induction of plant immune responses to pathogen infection.
(TIF)

Table S1 **List of bacteria used in this study.**
(TIF)

Table S2 **List of primers used in this study.**
(TIF)

Table S3 **List of plasmids used in this study.**
(TIF)
