# Supplementary figures and images for: SEC14 Phospholipid Transfer Protein Is Involved in Lipid Signaling-Mediated Plant Immune Responses in Nicotiana benthamiana
Source: PLoS One. 2014 May 20;9(5):e98150. doi: 10.1371/journal.pone.0098150 (PMC4028302; doi:10.1371/journal.pone.0098150)

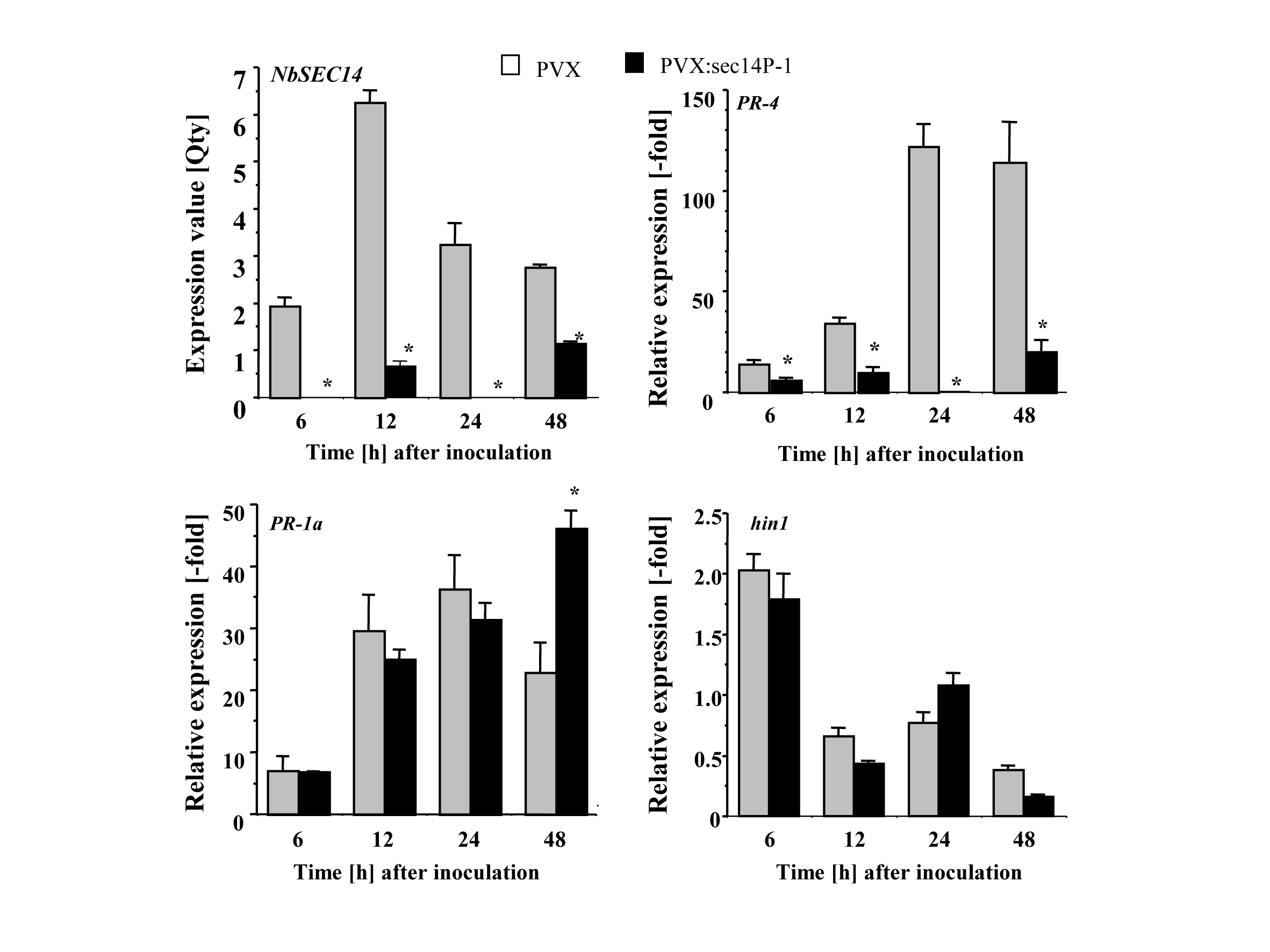

Supplement: Figure S1 — Alignment of NbSEC14 with its homologues in N. benthamiana genome, and virus-induced gene silencing of NbSEC14 . (a). Alignment of NbSEC14 with its homologues in N. benthamiana genome. cDNA fragments used for VIGS experiments are shown in gray boxes (Sec14P1) and black box (Sec14P2). Bold characters with underlines show primer sequences used for qRT-PCR (secrtpF and secrtpR). Dashed lines show nucleatide sequences that are not presented. (b) VIGS of NbSEC14 with Sec14P2 cDNA, and the effect of NbSEC14 on NbSEC14 and PR-4 expression by inoculation with avirulent R. solanacearum. Asterisks denote values significantly different from PVX controls (*; P<0.05, t-test). (TIF) [file pone.0098150.s001.tif]

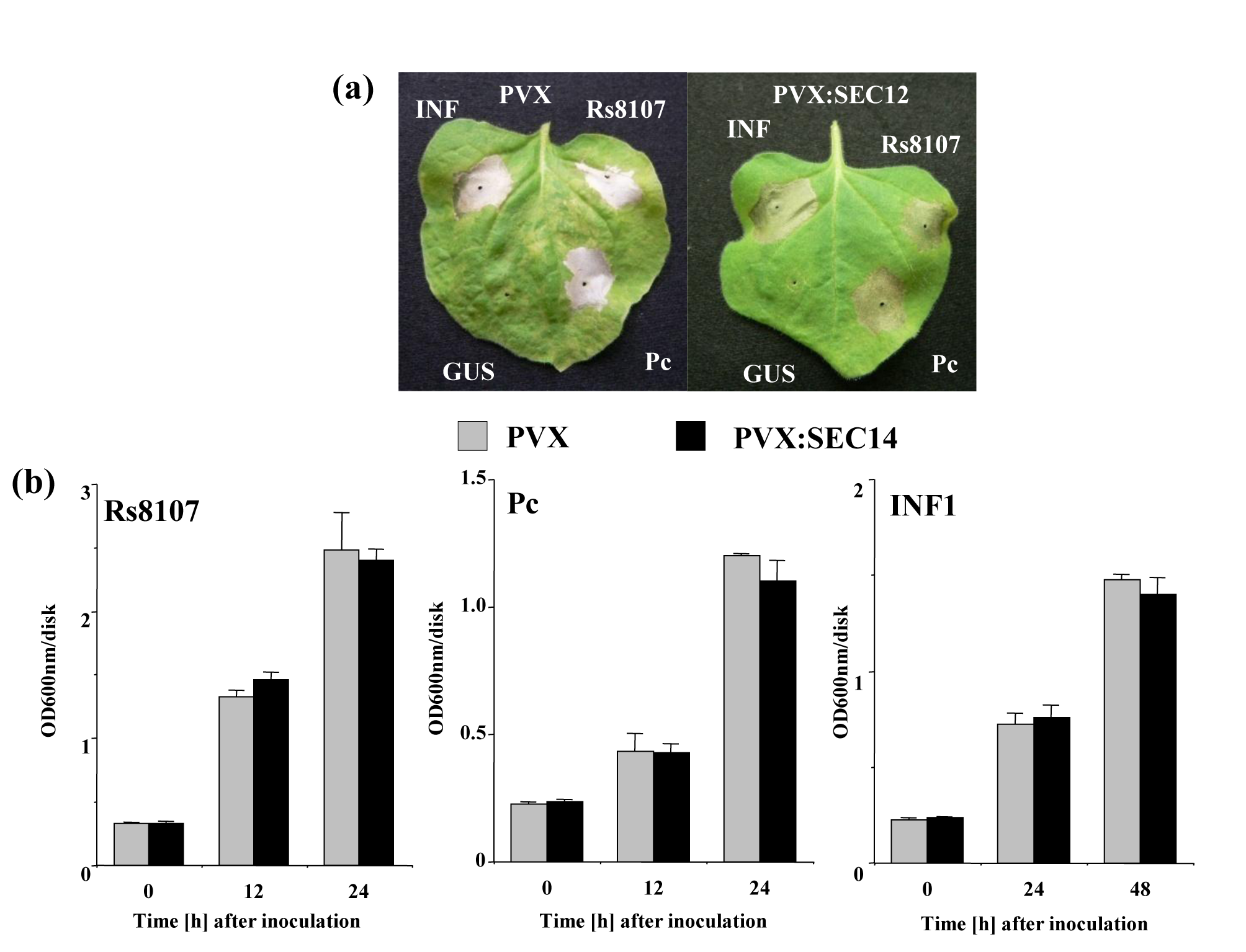

Supplement: Figure S2 — Effect of NbSEC14 -silencing on HR induction. Control and NbSEC14-silenced N. benthamiana plants were infiltrated with HR-inducible Rs8107, P. cichorii (Pc) or Agrobacterium harboring 35S-GUS (control GUS) or 35S-INF1 (INF1). (a) Pictures of N. benthamiana leaves taken 4 day after infiltration with each bacterium. (b) Control (Control; gray box) and NbSEC14-silenced (VIGS; back box) N. benthamiana plants were infiltrated with Rs8107, P. cichorii (Pc) or Agrobacterium harboring 35S-INF1 (INF1). Cell death was determined by Evans blue staining (OD600 nm disk−1). (TIF) [file pone.0098150.s002.tif]

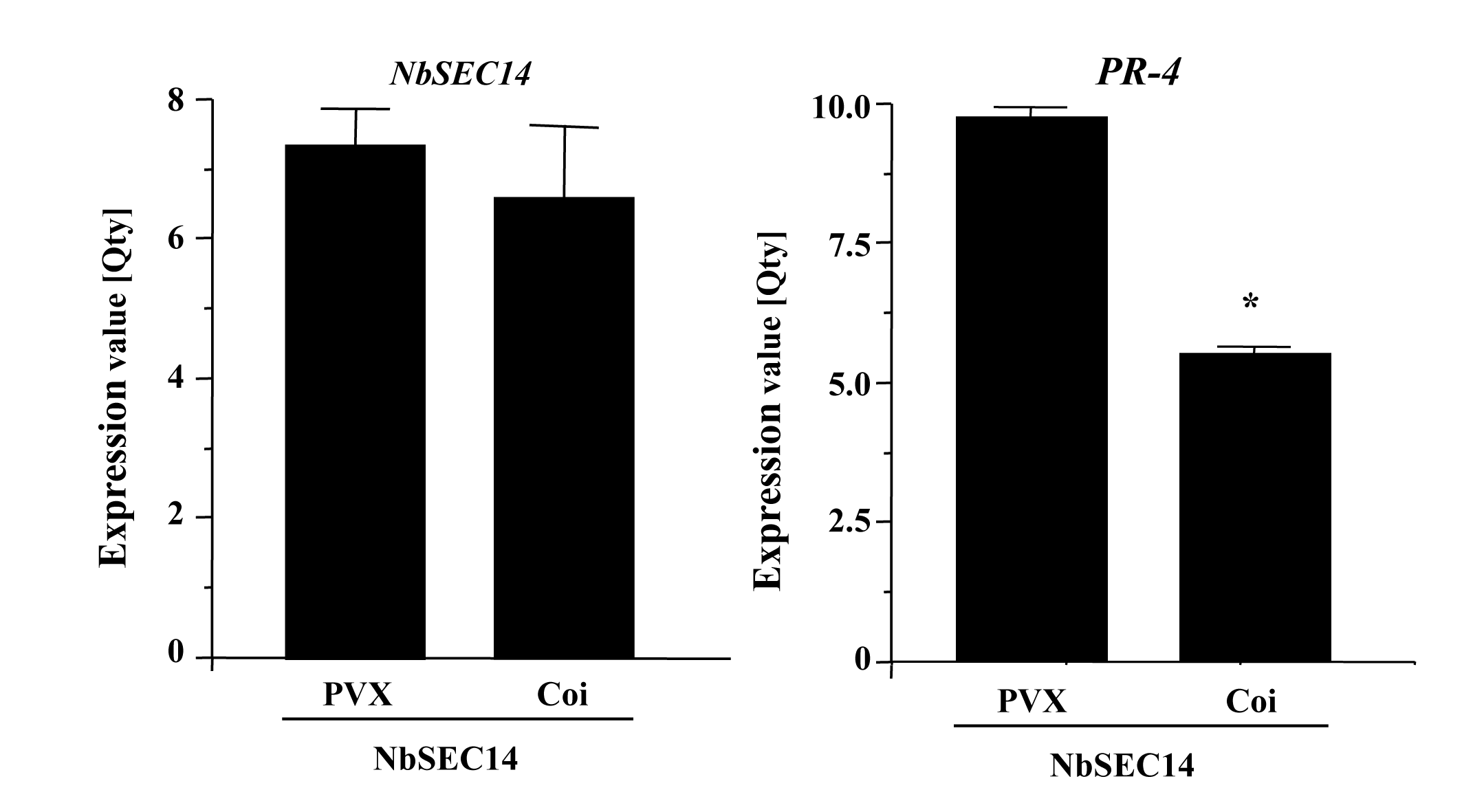

Supplement: Figure S3 — Role of jasmonic acid pathway in NbSEC14 -induced PR-4 genes expression. Total RNA was isolated from control (PVX), NbCoi1-silenced (Coi) N. benthamiana leaves inoculated with GUS and NbSEC14 expressing Agrobacterium. Relative expression of NbSEC14 and PR-4 transcripts were normalized with actin and calculated as relative to the GUS-expressing control. Values represent the means and SD from triplicate experiments. Asterisks denote values significantly different from empty vector (PVX)-expressing controls (*; P<0.05). (TIF) [file pone.0098150.s003.tif]

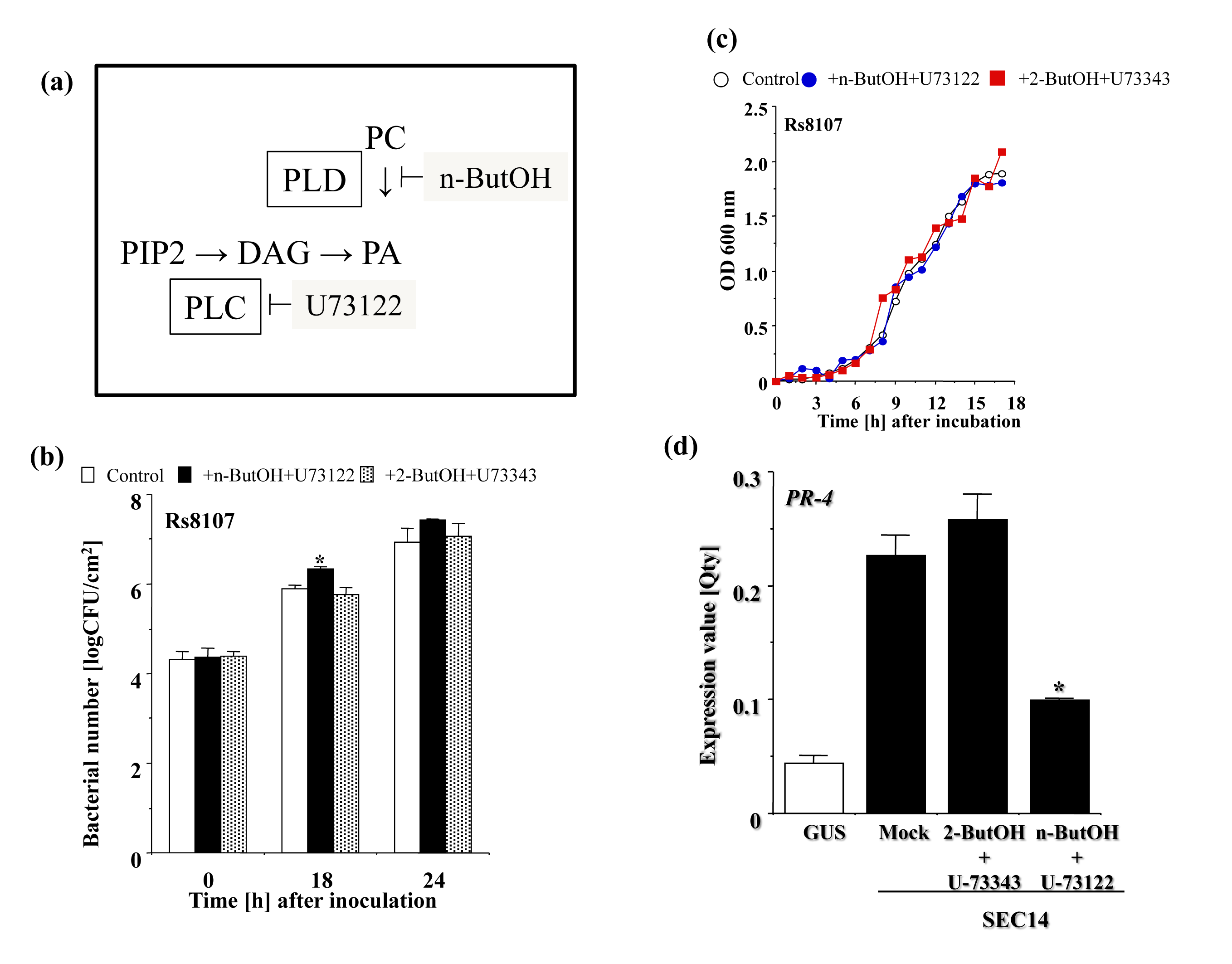

Supplement: Figure S4 — PLC and PLD activity is required for defense responses in N. benthamiana . (a) Schematic of phospholipase-dependent reactions in plant cells. (b) N. benthamiana leaves were infiltrated with Rs8107 (108 CFU ml−1) in the absence (Mock) or concomitant presence of 50 µM 1-[6-[((17β)-3-Methoxyestra-1,3,5[10]-trien-17-yl)amino]hexyl]-1H-pyrrole-2,5-dione (U73122; PLC inhibitor) and 0.1% normal-butanol (n-ButOH; PLD inhibitor) or 50 µM 1-[6-((17b-3-Methoxyestra-1,3,5(10)-trien-17-yl)amino)hexyl]-2,5-pyrrolidinedione (U73343) +0.1% 2-butanol (2-ButOH)(inactive analogue) (Kirik and Mudgett 2010), and bacterial population was determined by plating at specified time points. Values are means of four replicate experiments with SD. Asterisks denote values significantly different from control palnts (*; P<0.05). (c) Effect of inhibitors on in vitro growth of Rs8107. The bacteria were cultured in PY medium in the absence or presence of inhibitors (50 µM U73122+0.1% n-ButOH or 50 µM U73343+0.1% 2-ButOH). There was no obvious toxicity to bacterial growth. (d) Effect of inhibitors on PR-4 expression by NbSEC14-expression in N. benthamiana. GUS (GUS) or NbSEC14-expressing Agrobacterium (SEC14) were infiltrated in the absence (Mock) or presence of inhibitors into N. benthamiana. Expression values of PR-4 is expressed as [Qty] after normalization with actin. Values represent the means and SD from triplicate experiments. Asterisks denote values significantly different from GUS-expressing controls (*; P<0.05). (TIF) [file pone.0098150.s004.tif]

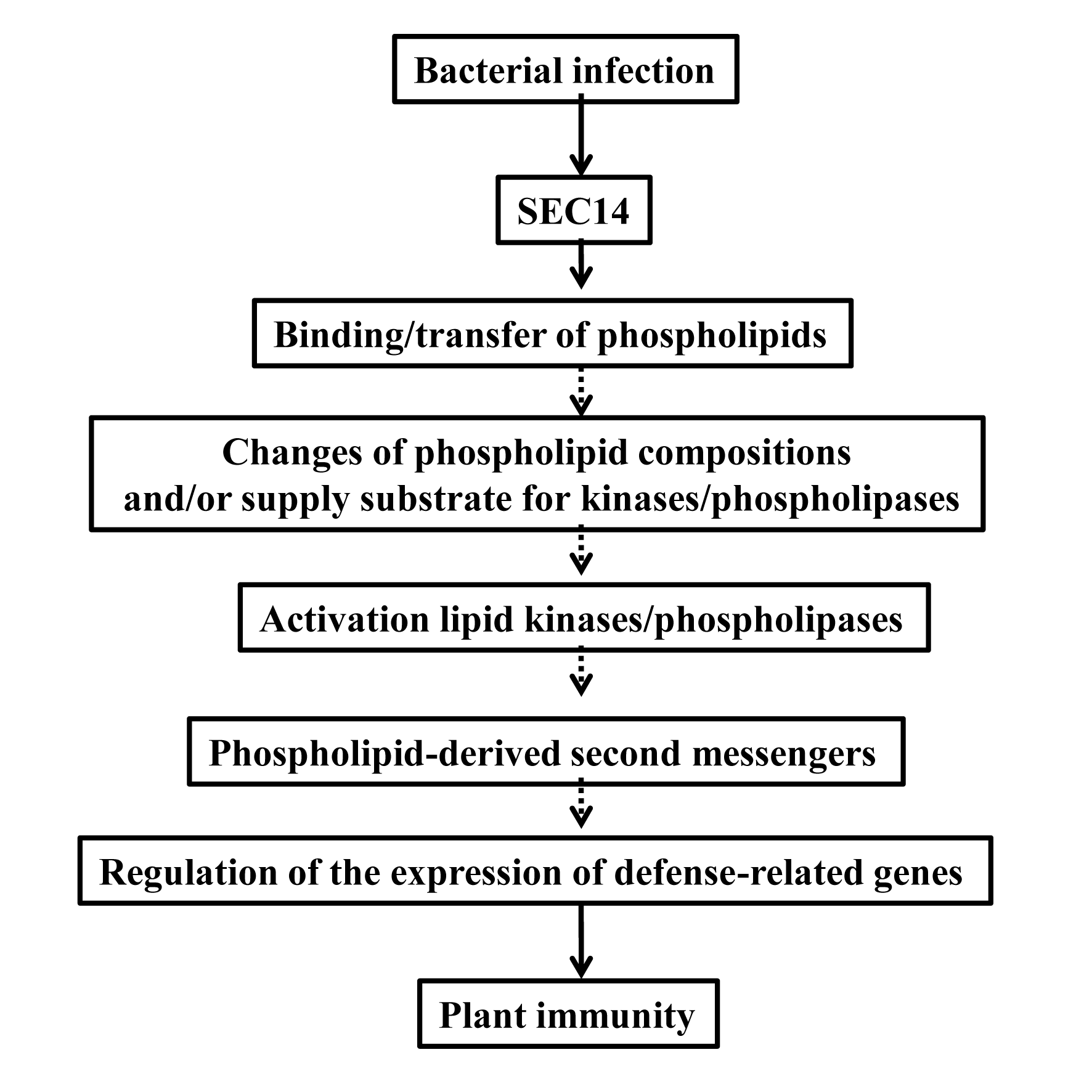

Supplement: Figure S5 — Function of NbSEC14 protein in the induction of plant immunity. NbSEC14 is expressed in response to bacterial infections. NbSEC14 protein initiates the binding/transfer of phospholipids, leading to changes in membrane lipid composition and substrate supply for lipid kinases and/or phospholipases. The subsequent generation of phospholipid-derived second messengers regulates other defense-related genes and the induction of plant immune responses to pathogen infection. (TIF) [file pone.0098150.s005.tif]

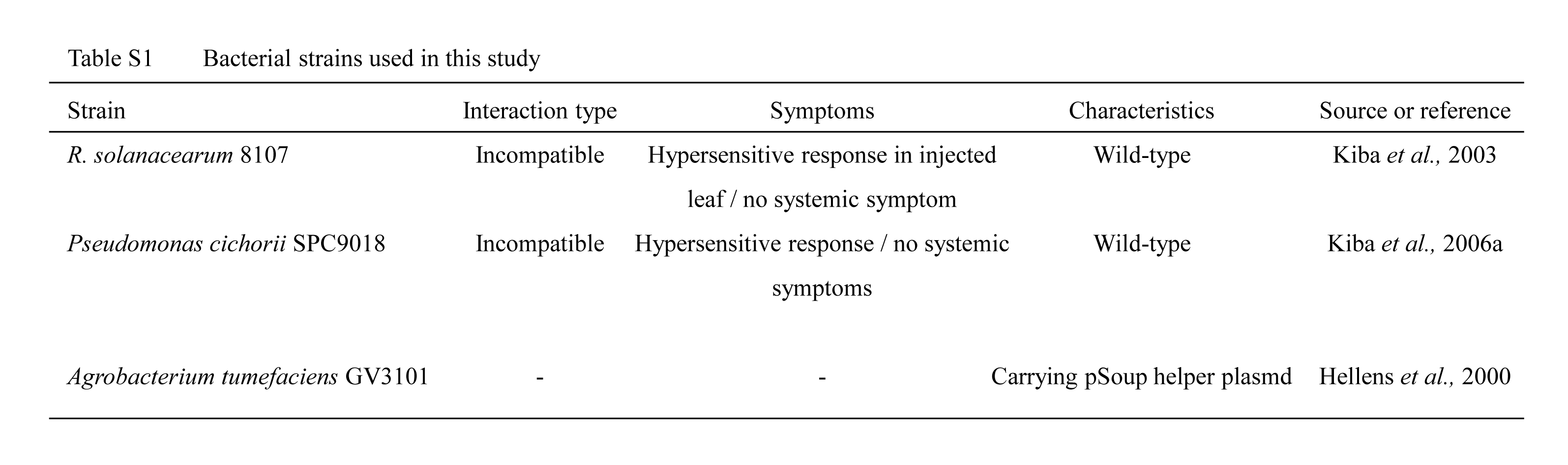

Supplement: Table S1 — List of bacteria used in this study. (TIF) [file pone.0098150.s006.tif]

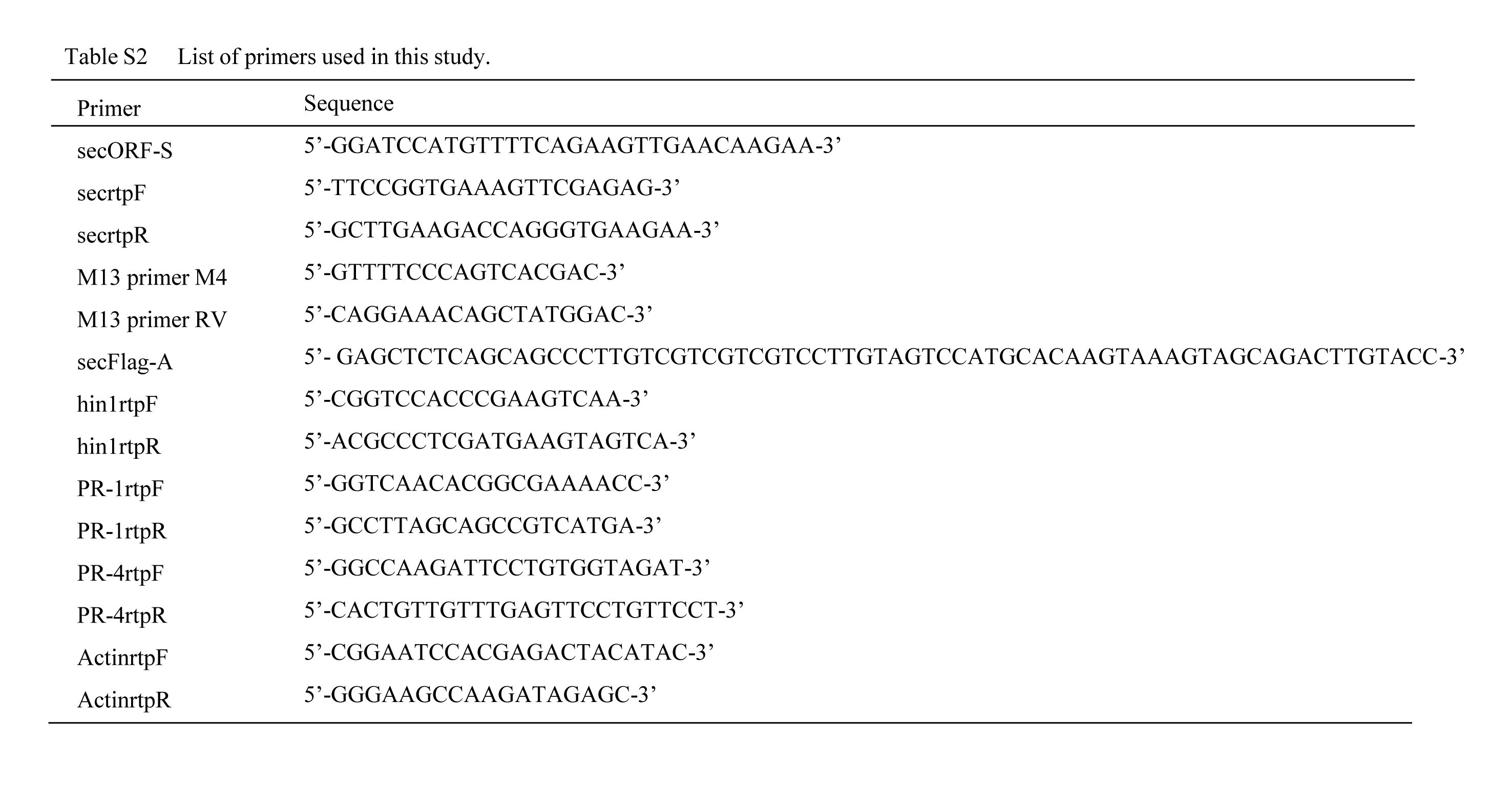

Supplement: Table S2 — List of primers used in this study. (TIF) [file pone.0098150.s007.tif]

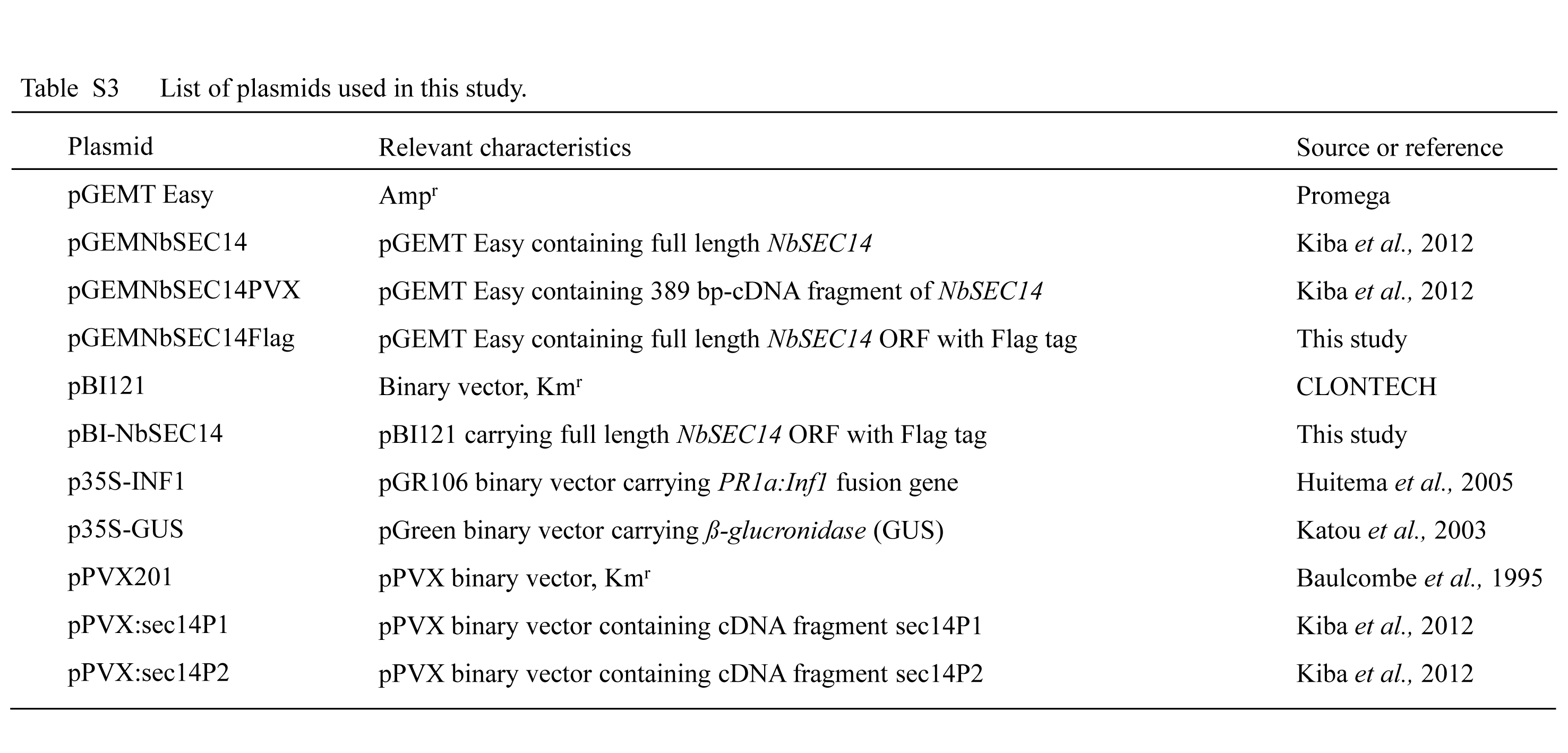

Supplement: Table S3 — List of plasmids used in this study. (TIF) [file pone.0098150.s008.tif]
